# Osteocytes function as biomechanical signaling hubs bridging mechanical stress sensing and systemic adaptation

**DOI:** 10.3389/fphys.2025.1629273

**Published:** 2025-07-15

**Authors:** Ma Yuze, Jin Hu, Lv Jun, Xu Cheng, Xin Tianwen, Zhang Junqiang

**Affiliations:** Department of Orthopedics, The Affiliated Suzhou Hospital of Nanjing Medical University, Suzhou Municipal Hospital, Gusu School, Nanjing Medical University, Suzhou, China

**Keywords:** osteocyte, crosstalk, mechanotransduction, mechanosensor, endocrine

## Abstract

Osteocytes, the most abundant bone cells embedded within mineralized matrix, are pivotal regulators of skeletal and systemic homeostasis. Recent advances highlight their mechanotransductive roles via mechanosensors, enabling detection of mechanical stimuli and conversion into biochemical signals to orchestrate bone remodeling. Beyond bone, osteokines derived from osteocytes engage themselves in bidirectional crosstalk with distant organs or tissues—modulating brain, liver, kidney, muscle, adipose tissue, nerve, blood vessel, and cancer. Hormonal and metabolic effects further integrate osteocyte activity into systemic regulation, while pathologies like diabetes or mechanical unloading disrupt their viability and signaling. Emerging evidence positions osteocytes as central hubs in interorgan networks, with neuron-like morphology enhancing their mechanosensing and communicative capacity. Understanding osteocyte-centric regulatory axes offers novel insights into bone-related diseases and systemic homeostasis.

## 1 Introduction

Osteocytes, as a critical population of terminally differentiated cells within skeletal tissue, have historically been underappreciated in bone research. This oversight may be attributed to their terminal differentiation status, which led researchers to perceive them as more quiescent and metabolically stable compared to dynamic bone-remodeling cells like osteoblasts and osteoclasts. Additionally, their unique anatomical localization embedded deeply within the mineralized bone matrix presents substantial technical challenges for experimental investigation. Nevertheless, recent years have witnessed a surge in osteocyte-focused studies employing multidisciplinary approaches, establishing these cells as a core player in bone histology. Contemporary research substantiates osteocytes’ central role in bone homeostasis modulation via their mechanotransductive competence and newly recognized endocrine functions in systemic physiological regulation.

## 2 Osteocyte biology of mechanical perception

Osteocytes, constituting 90%–95% of all cells in adult human bone, represent the most abundant cell type in skeletal tissue (Qin et al., 2020). These cells originate from mature, matrix-producing osteoblasts and constitute the terminal differentiation stage of the osteoblast lineage. Approximately 5%–20% of matrix-producing osteoblasts differentiate into late-stage osteocytes. While secreting the matrix to embedding themselves, osteoblasts undergo significant functional and morphological changes (Piemontese et al., 2016). As they become embedded into the matrix, they develop cellular extensions – precursor dendritic processes of mature osteocytes – that dynamically extend and retract until establishing proper connections with adjacent osteocytes (Zhang and Chen, 2023). Subsequently, these dendrites appear to anchor to neighboring cells while initiating mineralization processes that ultimately encase the cell within a hydroxyapatite lacuna. Upon completion of this mineralization, the embedded cell becomes functionally integrated into the lacunocanalicular network.

It is well established that mechanical loading serves as a critical external regulatory factor in bone remodeling processes (Cunningham et al., 2023). Osteocytes, constituting the most abundant cell type within bone tissue, demonstrate a fundamental relationship with mechanical loading that remains central to contemporary osteocyte research. Several studies have demonstrated that osteocytes exhibit mechanoresponsive characteristics, wherein mechanical unloading elicits apoptosis while mechanical loading counteracts apoptotic processes (Aguirre et al., 2006; Tan et al., 2006; Cabahug-Zuckerman et al., 2016). Furthermore, mechanical unloading induces site-specific and loading-specific modifications in osteocyte morphology and function, as evidenced by recent investigations (Metzger et al., 2020). These studies demonstrate that osteocytes are critically regulated by mechanical loading.

**FIGURE 1:**
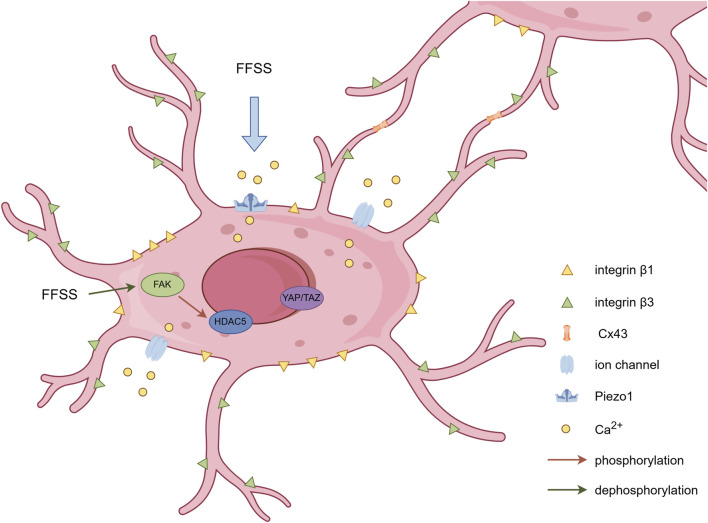
Schematic representation of mechanosensors in osteocytes. Dendritic processes establish intercellular connectivity via Cx43-containing gap junctions. Integrin β1 forms conventional focal adhesion complexes at osteocyte cell bodies, while integrin β3 colocalizes with specialized channel proteins along osteocyte dendritic processes. FFSS triggers calcium influx through Piezo1 and other ion channels on the osteocyte membrane, alters FAK phosphorylation status, and subsequently influences nuclear translocation of associated proteins. By Figdraw.

The mechanical signals that elicit responses in osteocytes primarily include two types: mechanical deformation of the perilacunar bone matrix and fluid flow shear stress (FFSS). However, the macroscopic deformation of bone is relatively small compared to the microscopic stimuli. Relevant studies indicate that the mechanical strain required for mechanotransduction ranges from 1,000 to 5,000 με (Robling and Turner, 2002), while the minimum strain needed to initiate an osteogenic response at the cellular level is of the order of 10,000 με (Robling and Turner, 2009). Several studies have demonstrated that osteocytes exhibit greater responsiveness to analytically estimated levels of fluid shear stress than to direct mechanical stretching applied at macroscopic strain levels measured *in vivo* (Fritton and Weinbaum, 2009; Hu et al., 2015; Ganesh et al., 2020; Sang and Ural, 2023). FFSS enables osteocytes to perceive mechanical stimuli and mount biological responses via mechanosensors. The following section will provide a review of several extensively investigated mechanosensors in osteocytes that have garnered significant research attention in recent years (Figure 1).

### 2.1 Processes of osteocytes

As mentioned above, there was a paradox that tissue-level strains are too small to initiate intracellular chemical responses directly. Han et al. developed a computational mechanobiology model of osteocyte mechanostransduction by using the ultrastructural data for the cell process cytoskeleton, the tethering elements that attach the processes to the canalicular wall and their finite flexural rigidity (Han et al., 2004). Their simulations demonstrated that processes with tethering elements attached enable strain amplification (Han et al., 2004). Furthermore, the dendritic processes exhibit preferential mechanosensitivity compared to cell bodies. Mechanical responsiveness (specifically to piconewton-level forces) is localized to osteocyte processes with attachment sites, while neither the cell body nor unattached processes demonstrate comparable mechanosensitivity (Thi et al., 2013). Another study showed that cell bodies can respond to mechanical loading from dendritic processes, whereas dendritic processes lack reciprocal responsiveness to somatic mechanical stimuli (Burra et al., 2010). Crucially, dendritic mechanosensation requires strong integrin-based adhesion to the pericellular glycocalyx, revealing an integrin-dependent mechanism for force transduction.

### 2.2 Integrin-mediated focal attachments of osteocytes

Integrin-mediated focal attachments serve as critical mechanosensors in osteocytes. Qin et al. summarized that integrin β1 forms conventional focal adhesion complexes at osteocyte cell bodies, while integrin β3 colocalizes with specialized channel proteins along osteocyte dendritic processes (Qin et al., 2020). They further found that the deletion of β1 or β3 integrin in osteocytes can both lead to severe low bone mass and compromised biomechanical properties in load-bearing bones, which is probably due to the destruction in cell morphology and microstructure (Qin et al., 2022; 2023). Furthermore, integrin β3 has been identified to interact with other crucial mechanosensitive membrane components (Cabahug-Zuckerman et al., 2018). In native osteocytes within their physiological microenvironment, critical membrane-associated proteins involved in mechanotransduction - including pannexin1, purinergic 2X7 receptor, and CaV3.2-1 channel subunits - demonstrate preferential spatial colocalization with β3 integrin-enriched focal adhesion complexes (Cabahug-Zuckerman et al., 2018). Integrin α5 demonstrates mechanosensitive characteristics too. Osteocyte-specific deletion of Integrin α5 leads to significant elevation in apoptotic osteocyte populations and impaired cortical anabolic adaptation to mechanical stimuli, characterized by attenuated bone formation rates and amplified osteoclastic resorption activity (Zhao et al., 2022). These studies demonstrate the essential roles of osteocyte integrin in regulating bone mass and mechanotransduction.

### 2.3 Gap junctions of osteocytes

Osteocytes utilize gap junctions to maintain communication with neighboring cells. Connexin 43 (Cx43) is the most prominent connexin observed in bone and osteocytes (Donahue, 2000). Cx45 and Cx26 are also expressed in bone, while the functions of Cx45 and Cx26 in osseous tissue remain unelucidated (Loiselle et al., 2013). Interestingly, minimal Cx45 expression was detected in osteocytes and remained unaltered in response to mechanical loading (Gluhak-Heinrich et al., 2006). Mechanical stimulation on osteocytes via FFSS triggers gap junction activation, dynamic Cx43 protein relocation, and Cx43 upregulation with prolonged mechanical loading (Cheng et al., 2001). Furthermore, mechanical loading-induced Cx43 overexpression of protein level exhibited significantly higher magnitude in osteocytes relative to other skeletal cell populations (Gluhak-Heinrich et al., 2006). CX43 has been mechanistically associated with additional mechanosensory components. Batra et al. demonstrated that FFSS stimulation upregulated active AKT levels, which subsequently induced phosphorylation of both CX43 and integrin α5, ultimately promoting their interaction (Batra et al., 2014). Furthermore, mice lacking Cx43 in osteocytes reveal critical functional consequences, including heightened apoptotic activity, accelerated endocortical bone degradation, and dysregulated periosteal ossification (Bivi et al., 2012). Conversely, pharmacological activation of Cx43 in osteocytes demonstrates therapeutic efficacy in mitigating disuse-induced osteopenia and mechanically deprived osteocyte apoptosis (Zhao et al., 2024). These findings collectively demonstrate Cx43’s essential role in mediating mechanochemical signaling networks that coordinate osteocyte survival and bone remodeling dynamics.

### 2.4 Ion channels of osteocytes

Mechanosensitive ion channels play an indispensable role in mediating osteocytic perception of biomechanical signals during early-stage mechanotransduction pathways. Similar to many other cell types, osteocytes exhibit different types of ion channels, including transient receptor potential channels (TRPV), voltage-sensitive calcium channels (VSCC) and Piezo1. However, knockout mice for several subtypes of TRPV do not exhibit reduced bone mass (Li et al., 2021). Furthermore, mice with germline deletion of the L-type VSCC Cav1.3 respond normally to mechanical loading (Li et al., 2010). These findings collectively suggest that TRPVs and VSCCs may not be involved in sensing mechanical stimuli. Piezo1, a promising mechanogating ion channel, was reported to be required for changes in gene expression induced by FFSS in cultured osteocytes (Li et al., 2019). Conditional deletion of Piezo1 in osteocytes notably reduced bone mass and strength in mice (Li et al., 2019). Moreover, specific deletion of Piezo1 in mature osteocytes elicited marked decreases in bone mineral density, reduced trabecular bone volume, and attenuated cortical bone thickness (Li et al., 2025). The investigations above demonstrate that Piezo1 functionality in osteocytes plays an essential role in basal skeletal homeostasis.

### 2.5 Mechanotransduction of osteocytes

The preceding sections outlined several critical mechanosensory receptors in osteocytes. Nevertheless, how osteocytes perceive mechanical stimuli and initiate corresponding biological responses remains a key question. Activation of ion channels such as Piezo1 induces intracellular calcium influx, which subsequently initiates downstream calcium-dependent signaling cascades to drive biomechanical adaptations. Lewis et al. systematically investigated *in vivo* calcium dynamics through an innovative methodology, which combines a three-point bending apparatus for controlled mechanical loading of murine bones with real-time monitoring using genetically encoded fluorescent calcium indicators (Lewis et al., 2017). Their findings revealed that the proportion of responsive osteocytes, rather than calcium intensity levels, exhibited significant strain-dependent augmentation under physiological loading conditions (Lewis et al., 2017). Furthermore, integrin-mediated focal adhesion kinase (FAK) participates in mechanotransduction pathways, as evidenced by recent findings (Chen et al., 2023). Experimental investigations by Sato et al. demonstrated that FFSS induces FAK dephosphorylation, consequently leading to histone deacetylase 5 (HDAC5) phosphorylation at tyrosine 642 residue and subsequent nuclear translocation (Sato et al., 2020). Mechanical loading also stimulates osteocytes to generate secondary messengers including nitric oxide (NO), adenosine triphosphate (ATP), and prostaglandin E2 (PGE2), while simultaneously promoting YAP/TAZ nuclear translocation (Yan et al., 2020). These coordinated events ultimately modulate the transcription of mechanoresponsive genetic elements. Researchers found that mechanical loading induces transcriptional regulation of specific gene clusters classified as mechanosensitive osteocyte signatures (Zarka et al., 2021; Zhou et al., 2022). The mechanical stress-induced alterations in transcriptional profiles of osteocytes not only modulate cellular homeostasis but more importantly may also regulate the production of osteocyte-derived secretory mediators (Zheng et al., 2023), which will be addressed in subsequent sections.

## 3 Osteocyte biology of intercellular communication

**FIGURE 2:**
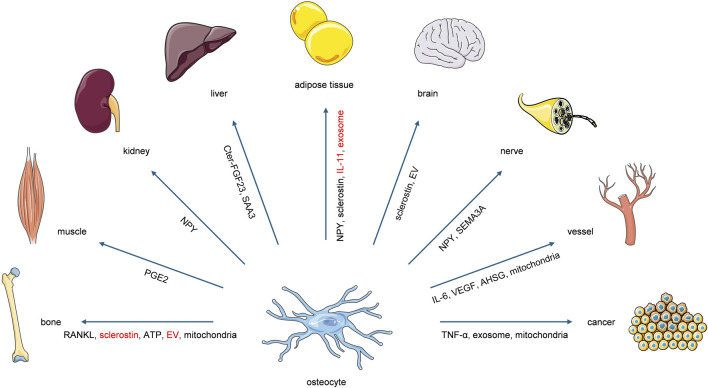
Osteocytes exert systemic regulatory effects on bone and extraskeletal organs or tissues through the secretion of diverse molecular factors. The factors highlighted in red represent those reported to exert regulatory functions under the modulation of mechanical loading. Notably, these highlighted factors lack direct evidence of mechanical-loading modulation while functioning in other organs, which requires further verification.

Recent studies have established osteocytes as active contributors to whole-body systemic regulation. This paradigm shift indicates that osteocytes not only receive systemic regulatory inputs from metabolic processes and distant organs or tissues, but also function as initiators of reciprocal regulatory feedback, particularly within mechanical loading microenvironments. The forthcoming sections will methodically expound the osteocyte-orchestrated intercellular communication networks, with specific focus on their modulatory functions directed toward extraskeletal organs and tissues (Figure 2; Table 1).

**TABLE 1:** Summary of osteocyte-mediated regulation.

Target	Factor	Mechanical stimulation	Effect	References
Bone	RANKL	No	Promote bone resorption	Nakashima et al. (2011) Xiong et al. (2011) Xiong et al. (2015) Xiong et al. (2018)
Bone	Sclerostin	Yes⬇	Suppress bone anabolism	Sapir-Koren and Livshits (2014) Li et al. (2008)
Bone	ATP	No	Induce osteocyte RANKL expression	McCutcheon et al. (2020)
Bone	EV	Yes⬆	Promote bone anabolism	Morrell et al. (2018)
Bone	EV	No	Young osteocyte-derived EVs promote osteogenesis	Wang et al. (2024)
Bone	Mitochondria	No	Restore metabolic function of osteocytes	Gao et al. (2019)
Muscle	PGE2	No	Stimulate myoblast proliferation	Mo et al. (2015)
Kidney	NPY	No	Contradictory results	Tan et al. (2023) Zoccali et al. (2018)
Liver	Cter-FGF23	No	Regulate hepcidin production	Agoro et al. (2021) Courbon et al. (2023)
Liver	SAA3	No	Impair cholesterol metabolism	Huang et al. (2024)
Adipose tissue	NPY	No	Promote adipocyte differentiation	Zhang et al. (2021)
Adipose tissue	Sclerostin	No	Promote adipocyte differentiation	Kim et al. (2017) Kurgan et al. (2022)
Adipose tissue	IL-11	Yes⬆	Inhibit adipogenesis	Kido et al. (2009) Dong et al. (2022)
Adipose tissue	Exosome	Yes⬆	Inhibit thermogenesis	Ma et al. (2024)
Brain	Sclerostin	No	Induce Aβ overproduction	Shi et al. (2024)
Brain	EV	No	Young osteocyte EVs ameliorate cognitive deficits	Jiang et al. (2022)
Nerve	NPY	No	Promote nociceptive neurite outgrowth	Qian et al. (2024)
Nerve	SEMA3A	No	Guide axon growth	Hayashi et al. (2019)
Endothelium	IL-6	No	Facilitate osteoclast precursor adhesion	Cheung et al. (2012)
Endothelium	VEGF	No	Promote angiogenesis	Prasadam et al. (2014)
Endothelium	AHSG	No	Modulate vascular calcification	Mattinzoli et al. (2016)
Endothelium	Mitochondria	No	Maintain TCV integrity	Liao et al. (2024)
Cancer	Exosome	No	Confer chemoresistance	Cheng et al. (2023)
Cancer	TNF-α	No	Suppress cancer proliferation	Verbruggen et al. (2024)
Cancer	Mitochondria	No	Inhibit cancer development	Zhou et al. (2024)

Osteocytes exert systemic regulatory effects on bone and extraskeletal organs or tissues through the secretion of diverse molecular factors. The factors marked as “yes” in the “mechanical stimulation” category represent those reported to exert regulatory functions under the modulation of mechanical loading. Notably, the factors mentioned above designated as “no” currently lack direct evidence of mechanical-loading modulation while functioning in other organs, which requires further verification.

### 3.1 Osteocytes and systemic metabolism

Despite their deep embedding within mineralized matrix, osteocytes remain responsive to systemic influences. A notable example manifests through metabolism alterations. Multiple studies have identified detrimental effects of glucose metabolism dysregulation on osteocyte viability. In cortical bone specimens from type 1 diabetes mellitus (T1DM) patients, accelerated osteocyte apoptosis and lacunar mineralization were observed compared to age-matched controls, suggesting T1DM promotes skeletal aging and compromises bone biomechanical integrity (Dragoun Kolibová et al., 2023). However, experimental findings regarding diabetes-induced osteocyte morphological changes in murine models demonstrated conflicting outcomes across studies (Mabilleau et al., 2016; Yao et al., 2022). Notably, a separate investigation documented that sustained hyperglycemic conditions increased osteocyte cellularity and lacunar density (Ay et al., 2020). While considerable controversy persists regarding the microstructural impacts of glucose metabolism dysregulation on osteocytes, its detrimental effects on osteocyte physiology are well-established. *In vitro* experimentation has demonstrated that hyperglycemia impairs osteocyte mechanotransduction capacity (Maycas et al., 2017). Furthermore, type 2 diabetes mellitus (T2DM) was shown to compromise exogenous cyclic loading-induced improvements in bone architecture and biomechanical strength in murine models, a phenomenon associated with weakened calcium oscillatory dynamics of osteocytes (Shao et al., 2024). Similarly, chronic alcohol consumption induces osteocyte apoptosis accompanied by significant reductions in bone mineral density, trabecular thickness, and cortical bone thickness (Maurel et al., 2011). High-carbohydrate and high-fat dietary regimens disrupt osteocyte mitochondrial bioenergetics, exacerbate senescence-associated secretory phenotypes, and impair perilacunar/canalicular remodeling processes, ultimately deteriorating bone quality (Dole et al., 2024). These findings collectively establish the osteocytes integration into multidimensional systemic regulatory networks, highlighting their responsiveness to whole-body homeostatic modulation.

### 3.2 Osteocytes and hormones

Hormones, as critical regulatory agents, exert potent effects on maintaining osteocyte homeostasis. Substantial evidence confirms that parathyroid hormone and growth hormone play indispensable roles in preserving osteocyte viability and physiological functionality (Yajima et al., 2010; Liu et al., 2019). While long-term clinical use of glucocorticoid (GC) has been associated with detrimental effects on bone tissue, their precise cellular impacts on osteocytes remain incompletely characterized. The comprehensive results of the latest research indicate that GC treatment exerts dose-dependent effects on osteocytes, with lower concentrations activating autophagic pathways and higher doses promoting apoptotic mechanisms (Xia et al., 2010; Jia et al., 2011). Furthermore, GC is also interrelated with the regulation of osteocytes by mechanical loading. Mechanical loading has been demonstrated to counteract dexamethasone-induced osteocyte apoptosis (Kitase et al., 2010), while GC can suppress osteocyte-mediated perilacunar remodeling processes (Fowler et al., 2017). Estrogen exerts a more pronounced effect on osteocytes. Postmenopausal osteoporosis is a prevalent form of bone loss, and the resultant estrogen deficiency often induces osteocyte apoptosis and alters local bone microarchitecture. Estrogen has been shown to be associated with the capacity of osteocytes to detect subtle mechanical stimuli. Lewis et al. subjected the bones of ovariectomized (OVX) mice and those of control mice to mechanical loading experiments (Lewis et al., 2021). They found that the number of osteocytes exhibiting Ca^2+^ signaling in OVX mice was comparable to that in control mice at higher strain levels; however, under loads lower to 1,000 με, there was a dramatic reduction in the number of responding osteocytes (Lewis et al., 2021). Ovariectomy significantly modified the abundance, composition, and spatial organization of mechanosome complexes on osteocyte dendritic processes (Lewis et al., 2021). Furthermore, investigators observed that estrogen deficiency diminished Connexin 43 expression and hemichannel function (Ma et al., 2019). They found that impairment of Cx43 hemichannels in osteocytes caused by estrogen deficiency accelerates vertebral trabecular bone loss and increases osteocyte apoptosis (Ma et al., 2019). Interestingly, estrogen also induces the autocrine secretion of Semaphorin 3A (SEMA3A) by osteocytes. Estrogen stimulates osteocyte expression of Sema3A, which engages its receptor on osteocytes to promote their survival and preserve bone homeostasis (Hayashi et al., 2019). Loss of SEMA3A leads to pronounced decreases in bone mass (Hayashi et al., 2019). Moreover, estrogen confers protective effects on osteocytes by antagonizing glucocorticoid-mediated mechanisms (Gu et al., 2005; Florencio-Silva et al., 2018). The aforementioned investigations collectively underscore the pivotal role of hormonal regulation in osteocyte dynamics.

### 3.3 Osteocytes and bone

Beyond systemic regulation by metabolism and hormones, osteocytes engage in cellular-level communication. The most extensively studied interactions of osteocytes involve their regulatory roles over bone homeostasis. Osteocytes sense and transmit bone anabolic or catabolic signals to effector cells via their canalicular network. Tatsumi et al. demonstrated that mice with osteocyte ablation developed fragile bones characterized by intracortical porosity, microfractures, trabecular bone loss, and osteoblastic dysfunction (Tatsumi et al., 2007). Soluble molecules secreted by osteocytes are widely recognized as primary mediators of osteoblast and osteoclast regulation. Scientific evidence confirms that osteocytes serve as a major source of receptor activator of nuclear factor-κB ligand (RANKL), with osteocyte-derived RANKL being a critical contributor to bone remodeling and disuse-induced bone loss (Nakashima et al., 2011; Xiong et al., 2011). Further supporting this, Xiong et al. generated mature osteocyte-specific RANKL knockout mice, revealing a high-bone-mass phenotype in the conditional knockout models (Xiong et al., 2015). However, studies indicate that soluble RANKL deficiency does not alter bone mass or structure in growing mice but reduces osteoclast numbers while increasing cancellous bone mass in adult mice (Xiong et al., 2018), indicating a different regulation way for soluble RANKL.

In addition to the classical RANKL signaling, sclerostin has emerged as a prominent osteocyte-derived regulatory factor in recent years. Sclerostin expression is mechanosensitive, being suppressed under mechanical loading and upregulated during unloading (Sapir-Koren and Livshits, 2014). Li et al. developed sclerostin knockout murine models and observed a marked elevation in bone mineral density (BMD) and bone volume, primarily attributed to enhanced osteoanabolic activity (Li et al., 2008). Further investigations by Tu et al. utilizing osteocyte-specific sclerostin transgenic mice demonstrated that mechanical loading-induced anabolic effects necessitate sclerostin downregulation (Tu et al., 2012). The above studies indicate that the regulatory effect of sclerostin is closely related to the mechanical loading on osteocytes.

Beyond the famous osteokines mentioned above, osteocytes also release diverse molecular factors that participate in the homeostatic regulation of bone. McCutcheon et al. identified a novel osteocyte communication mechanism using a multiscale microfluidic system (McCutcheon et al., 2020). Their work confirmed that apoptotic osteocytes release ATP through Panx1 channels, which subsequently induces RANKL expression in adjacent osteocytes and initiates bone remodeling (McCutcheon et al., 2020). Furthermore, osteocyte apoptosis is mechanistically linked to osteoclast activation. Studies revealed that apoptotic osteocytes secrete interleukin-6 (IL-6) and soluble IL-6 receptor, upregulating endothelial ICAM-1 expression to facilitate osteoclast precursor adhesion to vascular endothelia (Cheung et al., 2012). However, Bakker et al. reported that mechanical stimulation of osteocytes amplifies IL-6 production, redirecting osteocyte signaling toward osteoblasts (Bakker et al., 2014). Notably, IL-6 exhibits complex and context-dependent effects on both osteoblast and osteoclast activities.

Osteocytes utilize other specialized regulatory pathways in addition to the aforementioned soluble molecular factors. Studies reveal that extracellular vesicles (EVs) released by osteocytes exert significant regulatory effects. Morrell et al. demonstrated that mechanical stimulation on osteocytes activates the production and release of EVs containing bone-regulatory proteins, thereby promoting osteoanabolic activity (Morrell et al., 2018). Wang et al. observed that young osteocyte-derived EVs (YO-EVs) enriched with tropomyosin-1 markedly enhance alkaline phosphatase activity, mineralization deposition, and osteogenesis-related gene expression in bone marrow stromal cells (BMSCs), while senescent osteocyte-derived EVs (SO-EVs) exhibit contrasting effects (Wang et al., 2024). These studies profoundly demonstrate the significant regulatory role of EVs derived from osteocytes. Additionally, Taylor et al. identified that direct physical contact by gap junctions between osteocytes and osteoblasts can also mediate the transmission of anabolic signaling (Taylor et al., 2007). Moreover, Gao et al. have demonstrated that healthy osteocytes possess the capability to donate mitochondria to neighboring osteocytes under metabolic stress conditions, thereby facilitating functional recovery of cellular metabolism (Gao et al., 2019). The aforementioned studies demonstrate that osteocytes play a central role in mechanical loading-mediated regulation of bone homeostasis and employ multiple mechanisms to facilitate intercellular communication.

### 3.4 Osteocytes and muscle

As integral components of the musculoskeletal system, muscles and bones collectively bear mechanical loading and exhibit intrinsic bidirectional regulatory mechanisms. Recent advancements have identified numerous muscle-derived factors, with irisin emerging as a key regulator demonstrating significant osteoprotective effects. Studies confirm that irisin induces notable enhancements in cortical bone mass and mechanical strength (Colaianni et al., 2015). Kim et al. demonstrated that irisin binds to osteocytes via αV integrins, concurrently enhancing osteocyte survival and promoting sclerostin secretion (Kim et al., 2018). Further investigations reveal that irisin suppresses mechanical unloading-triggered osteocyte apoptosis (Storlino et al., 2020). Additionally, irisin activates Unc-51 like autophagy activating kinase 1 (ULK1) signaling to induce osteocyte autophagy, thereby mitigating age-related osteoporosis (Li et al., 2024). Similarly, the muscle-secreted metabolite β-Aminoisobutyric acid (L-BAIBA) inhibits ROS-induced mitochondrial degradation in osteocytes, preventing bone loss (Kitase et al., 2018). Osteocytes reciprocally modulate muscle function. Osteocyte-derived PGE2 regulates myogenesis by binding to EP4 receptors, which stimulates myoblast proliferation (Mo et al., 2015). Emerging evidence from these investigations sheds light on osteocyte-muscle interactions, where the potent secretory activity of musculature implicates a broader spectrum of bidirectional regulatory signaling.

### 3.5 Osteocyte and kidney

Scientists previously hypothesized that renal regulation of skeletal homeostasis was primarily mediated through the kidney’s role in calcium and phosphate metabolism, with this effect being more closely associated with osteoblast and osteoclast activity. However, recent research has demonstrated that renal pathologies can directly compromise osteocyte viability and function. Hsu et al. demonstrated that chronic kidney disease (CKD) impairs mitophagy processes and mitochondrial homeostasis of osteocytes (Hsu et al., 2025). Furthermore, exposure to uremic toxins resulted in the accumulation of mitolysosomes and mitochondria exhibiting disrupted ultrastructure. CKD also promotes the significant downregulation of dentin matrix protein 1 (DMP1). Experimental evidence suggests that DMP1 supplementation in CKD models improves bone quality, suppresses fibroblast growth factor 23 (FGF23) overproduction, and attenuates associated cardiovascular complications (Zhang and Chen, 2023). Neuropeptide Y (NPY) derived from osteocytes exhibits renoprotective effects via Y1 receptor-mediated inhibition of NF-κB signaling, which mitigates M1 macrophage polarization and renal necroinflammatory responses during acute kidney injury (Tan et al., 2023). Paradoxically, a European CKD cohort study revealed that elevated NPY levels correlate with proteinuria, accelerated CKD progression, and heightened risk of end-stage renal disease (Zoccali et al., 2018). The aforementioned studies have preliminarily elucidated the mechanisms by which osteocytes regulate renal function independent of calcium and phosphate metabolism. However, current research on the reciprocal crosstalk between the kidney and osteocytes remains limited, which requires substantial further investigation.

### 3.6 Osteocytes and liver

As one of the largest metabolic organs, the liver maintains systemic interorgan communication. Cross-sectional studies on liver cirrhosis demonstrate correlations between disease progression and alterations in the osteocyte lacunar network along with serum sclerostin levels, with disrupted osteocyte lacunar architecture and elevated serum sclerostin concentrations in cirrhotic patients (Rhee et al., 2014; Jadzic et al., 2022). Similarly, a latest cross-sectional study revealed an increased proportion of mineralized osteocyte lacunae in male individuals with alcohol-associated liver disease (Jadzic et al., 2025). However, the specific hepatic-derived mediators influencing osteocytes remain undefined. Lecithin-cholesterol acyltransferase (LCAT), a liver-secreted enzyme, has recently been implicated in skeletal homeostasis. Lu et al. reported that LCAT deficiency exacerbates bone loss in murine models of hepatic osteodystrophy, while LCAT supplementation ameliorates liver fibrosis and enhances hepatic function by facilitating reverse cholesterol transport from bone to liver (Lu et al., 2022). The discovery of LCAT indicates the possibility of extensive regulation of a large number of liver-derived enzymes.

Conversely, osteocytes modulate hepatic activity through several osteokines. During inflammatory states, C-terminal fibroblast growth factor 23 peptides (Cter-FGF23) is found to suppress bone morphogenetic protein (BMP)-dependent hepcidin production in the liver, thereby influencing erythropoietin synthesis and systemic iron regulation (Agoro et al., 2021; Courbon et al., 2023). Huang et al. demonstrated that osteocytes produce serum amyloid A3 (SAA3), a secretory protein that specifically interacts with toll-like receptor 4 expressed on hepatocyte membranes, thereby impairing the hepatic capacity for cholesterol metabolism (Huang et al., 2024). These findings preliminarily delineate bidirectional liver-osteocyte crosstalk, though substantial mechanistic and interaction gaps persist, which need further investigation.

### 3.7 Osteocytes and adipose tissue

Osteocytes and adipose tissue, sharing a mesodermal origin and common differentiation precursors, exhibit developmental and functional interdependencies. Leptin, primarily secreted by adipocytes, serves as a key adipokine. Despite the presence of leptin receptors in osteocytes, studies indicate that leptin receptors (LepR) of osteocytes lack indispensable roles in normal skeletal maturation (Wee et al., 2022). However, constitutive signal transducer and activator of transcription 3 (STAT3) activation enables LepR in osteocytes to enhance cortical bone consolidation, manifested as increased proportions of hypermineralized bone matrix (Wee et al., 2022). These findings imply a restricted modulatory capacity of adipocytes toward osteocytes, underscoring the necessity for further mechanistic investigations to delineate this intercellular regulatory axis.

Conversely, osteocytes exert more profound regulatory effects on adipocytes. Experimental ablation of osteocytes in murine models unexpectedly induced complete depletion of white adipose depots, with phenotypic reversal upon osteocyte replenishment, underscoring osteocytes’ essential role in adipose tissue maintenance (Sato et al., 2013). Additionally, peroxisome proliferator-activated receptor-γ (PPARγ)-regulated osteocyte-derived factors such as BMP7 mediate bone-fat crosstalk. Mature osteoblast/osteocyte-specific PPARγ knockout mice exhibit age-dependent body composition shifts—reduced adiposity, increased lean mass, enhanced insulin sensitivity, and elevated energy expenditure (Brun et al., 2017). Under high-fat diet challenges, these mutants maintained glycemic stability via augmented adipose tissue browning, suppressed hepatic gluconeogenesis, and attenuated steatosis (Brun et al., 2017). Collectively, these studies indicate that osteokines may exert substantial regulatory effects on adipocyte biology.

Current studies have identified multiple osteokines participating in the regulatory network of adipocyte biology. Zhang et al. demonstrated that the NPY mentioned above exerts modulatory control over adipogenic differentiation (Zhang et al., 2021). Osteocyte-specific NPY deletion generates high-bone-mass phenotypes and mitigates aging- and ovariectomy-induced bone-fat imbalances, implicating NPY secretion in promoting adipocyte differentiation (Zhang et al., 2021). Similarly, sclerostin-deficient mice display not only substantial bone volume augmentation but also attenuated adipose tissue deposition, concomitant with enhanced insulin sensitivity (Kim et al., 2017). Furthermore, exercise-induced downregulation of circulating sclerostin levels correlates with diminished sclerostin content in white adipose depots and elevated β-catenin expression, suggesting β-catenin signaling as a potential mechanistic link between sclerostin and adipose regulation (Kim et al., 2017; Kurgan et al., 2022). Interleukin-11 (IL-11), a mechanoresponsive cytokine in bone, is upregulated by mechanical stimulation to promote osteogenesis while inhibiting adipogenesis (Kido et al., 2009). Dong et al. revealed that global IL-11 knockout models exhibit reduced bone mass, impaired mechanical loading-induced bone formation, and systemic metabolic perturbations including adiposity and glucose intolerance (Dong et al., 2022). Strikingly, osteoblast/osteocyte-specific IL-11 deletion recapitulated analogous phenotypes, whereas adipocyte-specific IL-11 ablation showed no pathological alterations, establishing osteocyte-derived IL-11 as a critical mediator of mechanical loading-dependent bone-fat crosstalk (Dong et al., 2022).

Brown adipose tissue (BAT), distinguished from white adipose tissue by its unique thermogenic capacity and distinct metabolic activity, exhibits functional correlations with skeletal homeostasis. Tian et al. demonstrated that cold exposure potentiates BAT thermogenic capacity while concomitantly diminishing bone mineral density (Tian et al., 2024). BAT functional impairment via surgical ablation or mitochondrial disruption intensifies hypothermia-associated osteopenia, highlighting BAT’s endocrine regulatory function in bone homeostasis maintenance (Tian et al., 2024). At the molecular level, cold-stimulated skeletal deterioration appears to trigger osteocyte apoptosis, while BAT-derived mediators may orchestrate bone remodeling through endocrine signaling pathways targeting osteocytes’ networks (Du et al., 2021). Collectively, these findings suggest that BAT activity regulates bone mass through secretory metabolites or BAT-derived cytokines. Conversely, osteocyte-mediated regulation of BAT has recently been elucidated. Ma et al. observed that mechanical unloading in mice induces concurrent bone loss and BAT activation (Ma et al., 2024). Further analysis revealed that mechanical loading promotes osteocyte exosome secretion enriched with let-7e-5p, whereas unloading suppresses this process. Additionally, they found that osteocyte-derived exosomes were shown to be able to reach BAT, exerting remote regulatory effects on its activity. These discoveries unveil a bidirectional regulatory axis between thermogenic BAT and osteocytes. To sum up, there is extensive mutual regulation between osteocytes and adipocytes.

### 3.8 Osteocytes and brain

For decades, bone and brain were considered functionally independent organs. However, emerging studies now reveal intricate crosstalk between these systems, particularly through osteocyte-neuronal interactions. Li et al. investigated behavioral effects of intracerebroventricular sclerostin administration in adult mice, demonstrating that acute intracerebral sclerostin elevation triggers anxiety-like phenotypes, diminishes social dominance, and reduces hippocampal pyramidal neuron dendritic complexity (Li et al., 2022). Shi et al. further delineated sclerostin’s role in Alzheimer’s disease (AD) progression (Shi et al., 2024). Their clinical data linked elevated serum sclerostin levels to exacerbated cognitive decline in AD patients independent of sex. Critically, osteocyte-derived sclerostin crosses the blood-brain barrier, mechanistically driving amyloid-β (Aβ) overproduction via β-catenin/BACE1 signaling, ultimately impairing synaptic plasticity and memory in aged mice (Shi et al., 2024). Jiang et al. identified osteocyte-derived extracellular vesicles (OCY-EVs) as systemic mediators of brain homeostasis (Jiang et al., 2022). Under physiological conditions, young osteocyte EVs ameliorate cognitive deficits and AD pathology, whereas aged osteocyte EVs lack therapeutic efficacy. Proteomic profiling demonstrates that young OCY-EVs are enriched with neuroprotective factors targeting AD pathways (Jiang et al., 2022). Collectively, these findings demonstrate the pleiotropic regulatory capacity of osteokines within cerebral physiology, while the potential involvement of mechanical loading in mediating such osteokine-brain signaling dynamics remains to be systematically investigated.

### 3.9 Osteocytes and nerve

Peripheral nerves were historically thought to primarily interact with the periosteum. However, emerging evidence demonstrates that osteocytes directly depend on peripheral innervation for functional support. Ma et al. found that muscarinic and nicotinic acetylcholine receptors are expressed in murine tibial tissues and MLO-Y4 osteocyte cell lines (Ma et al., 2014). Additionally, acetylcholine treatment markedly enhances osteocyte proliferation and viability, while regulating expression of NPY and reelin in MLO-Y4 cells. Elevated sympathetic tone may mechanistically correlate with osteopenic progression. Guo et al. demonstrated that sympathetic innervation exerts direct regulatory control over lacunar osteocyte-mediated cortical bone resorption, with heightened sympathetic tone observed during bone loss states (Guo et al., 2023). During lactational challenges, amplified sympathetic signaling induces osteocyte secretion of the neurotrophic factor netrin-1, thereby establishing an autocrine-amplified circuit that potentiates sympathetic nerve sprouting along cortical endosteal microenvironments. Gadomski et al. identified a cholinergic neuro-osteocyte regulatory interface governing osteogenesis and bone remodeling (Gadomski et al., 2022). They found that moderate mechanical loading enhances trabecular bone formation through this pathway, whereas cholinergic denervation compromises osteocyte viability and function, leading to osteopenia and defective adaptive responses to exercise. Osteocytes exhibit bidirectional neuromodulatory capabilities within peripheral neural circuits. Qian et al. demonstrated that osteoarthritis-associated osteocytes exhibit upregulated NPY secretion, which promotes nociceptive neurite outgrowth, thereby exacerbating osteoarthritis-associated chronic pain (Qian et al., 2024). Furthermore, SEMA3A derived from osteocytes may display axon-guidance regulatory functions (Hayashi et al., 2019). These collective findings delineate bidirectional neural-osteocyte crosstalk, yet the mechanistic underpinnings of osteocyte-derived neuromodulation warrant systematic exploration through interdisciplinary neuro-osteology approaches.

### 3.10 Osteocytes and blood vessel

The bidirectional regulatory axis between osteocytes and vascular system is one of the surprising discoveries in recent years. Osteocytes are implicated in both angiogenesis and pathological vascular calcification. Conditioned medium derived from MLO-Y4 osteocytes significantly enhanced endothelial cell proliferation, migratory capacity, and tubular network formation, concomitant with upregulated expression of angiogenesis-related genes, indicating osteocyte-derived paracrine factors as mediators of vascular remodeling (Prasadam et al., 2014). Mechanistic investigations revealed that MLO-Y4-secreted vascular endothelial growth factor (VEGF) activates the VEGFR2 signaling axis in human umbilical vein endothelial cells (UVECs), thereby promoting angiogenesis (Prasadam et al., 2014). The co-occurrence of vascular calcification and osteoporosis, a clinically paradoxical comorbidity termed the “calcification paradox” remains mechanistically enigmatic. Elevated serum sclerostin levels is found to correlate with the severity of vascular calcification (Qureshi et al., 2015). Moreover, Wang et al. revealed that EVs originating from senescent bone matrix preferentially promote adipogenesis of BMSCs over osteogenesis while exacerbating vascular smooth muscle cell calcification, which explains the “calcification paradox” to a certain extent (Wang et al., 2022). Additionally, Alpha-2-HS-glycoprotein (AHSG), a circulating glycoprotein predominantly synthesized by osteocytes, has recently been identified as a novel modulator of vascular calcification (Mattinzoli et al., 2016). The aforementioned investigations elucidate the regulatory effects of osteokines on vascular function.

Beyond secretory regulation, osteocytes modulate perivascular networks via specialized mitochondrial transfer mechanisms. Transcortical vessel (TCV) serves as critical conduits facilitating bidirectional exchange between the bone marrow vasculature and systemic circulation. Liao et al. found that osteocytes maintain TCV integrity through mitochondrial donation to endothelial cells, with partial osteocyte ablation inducing TCV regression (Liao et al., 2024). Moreover, genetic disruption of mitochondrial transfer via osteocyte-specific Rhot1 knockout similarly impaired TCV maintenance, while endothelial acquisition of osteocyte mitochondria rescued vascular dysfunction, demonstrating functional mitochondrial crosstalk (Liao et al., 2024). This distinctive discovery provides novel investigative perspectives for elucidating osteocyte-mediated vascular regulatory mechanisms.

### 3.11 Osteocytes and cancer

Bone, as a frequent site of tumor metastasis, exhibits a well-documented interplay between osteocytes and cancer cells. Multiple studies confirm that various cancer types exert cytotoxic effects on osteocytes, inducing osteocyte apoptosis (Pin et al., 2021; Jia et al., 2025). Intriguingly, breast cancer cells adaptively respond to mechanical stimuli, and alter osteocyte mechanosensing by increasing dendrite formation and downstream resorption (Wang et al., 2018). Conversely, osteocytes reciprocally regulate tumor behavior. Osteocyte-derived exosomal cargo has been shown to confer chemoresistance in multiple myeloma by inducing cancer stem cell-like phenotypic transitions (Cheng et al., 2023). Additionally, osteocyte-secreted tumor necrosis factor-alpha (TNF-α) suppresses cancer proliferation, though this effect is antagonized by tumor-derived transforming growth factor-beta (TGF-β) (Verbruggen et al., 2024). Surprisingly, Zhou et al. found that mitochondria transfer is also a way of regulation on cancer (Zhou et al., 2024). They demonstrated that osteocytes transfer mitochondria to metastatic cancer cells, thereby triggering the cGAS/STING-mediated antitumor response (Zhou et al., 2024). The research above presents a corner of bidirectional osteocyte-cancer crosstalk.

Mechanical stimuli further modulate this interaction. Low-magnitude, high-frequency vibration attenuates cancer extravasation by targeting osteocyte signaling, thereby inhibiting skeletal metastasis (Song et al., 2022). Fan et al. demonstrated stress magnitude-dependent duality: physiological loading induces mesenchymal-to-epithelial transition (MET) *in vitro* and suppresses osteolysis *in vivo*, whereas pathological overloading promotes epithelial-to-mesenchymal transition (EMT) and osteoclastic activation (Fan et al., 2020). These experimental findings underscore the multifaceted nature of mechanical stimulation effects. Analogously, distinct tumor cell types exhibit differential responses to biomechanical cues. Investigations revealed that osteocytes suppress metastatic progression in breast and prostate malignancies, manifesting attenuated proliferation and diminished invasive capacity in tumor cells (Verbruggen et al., 2021). Mechanical activation of osteocytes predominantly counteracts these tumor-suppressive effects in breast cancer models, whereas prostate cancer cells remain refractory to such biomechanical modulation (Verbruggen et al., 2021). The studies mentioned above focusing on osteocyte-tumor interactions, particularly those incorporating mechanical loading stimulation, provide critical insights into elucidating bone metastasis-associated mechanisms.

## 4 Discussion

In recent years, there has been a substantial increase in research focusing on osteocytes. The total number of osteocytes within the skeleton of an average adult human is estimated to be approximately 42 billion, interconnected via dendritic processes to form roughly 23 trillion connections, which facilitate signal transmission (Buenzli and Sims, 2015). In contrast, an adult human possesses approximately 100 billion neurons, which establish a total of about 1,500 trillion synapses (Budday et al., 2015). Consequently, it is evident that osteocytes and neurons exist on a similar order of magnitude in terms of cell number, and the connections they form serve analogous functions in communication. Despite this similarity, research related to neurons has long received a great deal of attention, while osteocytes remained comparatively understudied. Recent transcriptomic profiling has uncovered a unique transcriptome signature in osteocytes (Youlten et al., 2021). Youlten et al. identified 1,239 genes distinguishing osteocytes from other cell types, and 77% of these genes have no documented skeletal functions, yet show significant enrichment for neural network formation regulators (Youlten et al., 2021). The top 5 GO biological processes terms in early activation, which are upregulated in early osteocytes and remained expressed in mature osteocytes, are all associated with neurite outgrowth. For instance, as previously mentioned, osteocytes secrete Sema3A and netrin-1, which subsequently influence bone homeostasis. SEMA3A and netrin-1 belong to the Semaphorin and Netrin families, respectively, both of which are critically involved in neuron guidance. SEMA3A has also been reported to promote both total and average dendrite length in MLO-Y4 osteocyte-like cells (Niimura et al., 2016). Furthermore, netrin-1 induces directional elongation in osteocyte dendritic processes (Matsugaki et al., 2020). Other studies demonstrate that Sp7 deficiency in osteoblasts/osteocytes causes dendritic defects, and Sp7-dependent genes marking osteocytes are preferentially expressed in neurons (Wang et al., 2021). While some scientists propose this signature emphasizes the centrality of intercellular communication in osteocyte biology, we would suggest an alternative interpretation based on the above research: the striking morphological convergence between osteocytes and neurons may drive this transcriptional overlap, potentially reflecting shared cytoskeletal regulatory programs. Nevertheless, this neuron-like morphology undeniably serves dual biological purposes: it not only enhances osteocytes’ mechanosensing capacity for detecting mechanical loading, but also establishes an efficient communication network through interconnected cellular processes within the mineralized bone matrix. This evolutionary optimization suggests that the convergence with neuronal features may represent a fundamental biological strategy for coordinating multicellular responses to environmental stimuli.

The regulatory capacity of osteocytes is gaining increasing research attention. Groundbreaking work by Shi et al. has challenged conventional paradigms by demonstrating that osteocyte-derived sclerostin can breakthrough the blood-brain barrier (Shi et al., 2024). Furthermore, the synergy between osteocytes and the brain is astonishing during aging. On this basis, the establishment of the bone-brain axis with osteocytes as the core is highly anticipated. Osteocytes also have a special regulatory mechanism, namely mitochondrial transfer. On the one hand, osteocytes can transfer mitochondria to adjacent osteocytes (Gao et al., 2019); on the other hand, they can also transfer mitochondria to endothelial cells or cancer cells to exert regulatory functions (Liao et al., 2024; Zhou et al., 2024). In fact, the research on the function and transfer mechanism of mitochondria in osteocytes is gradually receiving attention, which is a key research direction in the future. However, significant gaps remain regarding the crosstalk between osteocytes and other organs. Firstly, the secretory profile of osteocytes has not been comprehensively characterized. Which factors are secreted by osteocytes under normal physiological conditions? Among these, which substances exhibit significant regulatory functions? Secondly, do bone-derived factors demonstrate specific targeting? Current research primarily focuses on whether bone-derived factors reach regions of interest—either through detecting increased levels in target organs or confirming their passage across biological barriers. Nevertheless, certain bone-derived factors (including exosomes) may potentially confer organ-specific targeting due to specialized surface modifications. The regulatory role of osteocytes under mechanical loading is fascinating. Normal stress stimulation maintains the normal secretion of osteocytes, while an increase or decrease (even microgravity) can greatly alter the secretion of osteocytes, thereby causing related changes throughout the body. At present, more research on this aspect is needed, especially the research on osteocytes as core cells regulating systemic homeostasis under microgravity.

In summary, osteocytes demonstrate the capacity to perceive and respond to mechanical loading stimuli while secreting signaling molecules that contribute to systemic regulation. With advancing research in this domain, osteocytes are likely to emerge as central regulatory elements within mechanical loading environments. We anticipate that future investigations focusing on osteocyte functionality will provide deeper mechanistic insights.
